# Shared Mechanisms of Alcohol and Other Drugs

**Published:** 2008

**Authors:** Maureen T. Cruz, Michal Bajo, Paul Schweitzer, Marisa Roberto

**Keywords:** Alcohol and other drug (AOD) use (AODU), AOD dependence, addiction, ethanol, cannabinoids, opioids, brain, γ-aminobutync acid (GABA), neurotransmitters, receptors, opioid system, cannabinoid system, amygdala, central nucleus of the amygdala (CeA), animal studies

## Abstract

Identifying the changes that occur in the brain as a result of alcohol and other drug (AOD) use is important to understanding the development of AOD addiction. The nerve cell signaling chemical (i.e., neurotransmitter) γ-aminobutync acid (GABA) plays an important role in the brain chemistry of addiction. Most drugs interact with binding molecules (i.e., receptors) for specific neurotransmitters and either block or facilitate binding at these receptors. Thus, cannabis and opiates act via receptors intended for internally derived (i.e., endogenous) cannabinoid and opiate substances. In contrast, alcohol does not appear to activate specific receptors. However, alcohol influences the activity of many transmitter systems including GABA and endogenous opioids and cannabinoids.

Alcohol and other drug (AOD) use creates a huge health burden for the United States and most countries worldwide and represents one of the most imposing medical and socioeconomic concerns for our society (Harwood et al. 2003; Kessler et al. 2005). Drug addiction comprises many complex behaviors in which an individual becomes increasingly preoccupied with obtaining drugs, leading to a loss of control over consumption and to the development of tolerance, dependence, and impaired social and occupational functioning (Hoffman and Tabakoff 1996; Koob et al. 1998). Current research aims to understand which specific nerve cells (i.e., neurons) and subcellular systems undergo molecular changes with drug exposure that lead to chronic drug abuse. This article will review our current understanding of how alcohol, opioids, and cannabinoids^1^ interact with the key substances in the brain, such as γ–aminobutyric acid (GABA), and with each other.

## Neuroadaptation

An important element in the development of drug addiction is the brain’s attempt to chemically counteract the influences of the drug (i.e., neuroadaptation). Neurobiological research on addiction focuses on uncovering the neuroadaptative mechanisms within specific brain circuits that mediate the transition from occasional and controlled drug use to loss of control over drug-seeking and drug-taking behaviors that define chronic addiction. In the last two decades, animal models have provided much information on the molecular mechanisms involved in the positive reinforcing effects of drugs. For example, such research has provided evidence implicating the neurotransmitter^2^ dopamine and the brain circuit through which it is transmitted (i.e., the mesocorticolimbic dopamine system) in the rewarding effects of AODs. More recently, specific components of the group of structures located near the bottom of the front of the brain (i.e., basal forebrain) have been implicated in the drug reward system and have been termed the “extended amygdala”^3^ (Koob 2003). The extended amygdala is composed of a group of structures, including the nucleus accumbens^4^ (NAcc), the central nucleus of the amygdala (CeA), and the bed nucleus of stria terminalis^5^ (BNST). The extended amygdala now is considered to play an important role in both the acute reinforcing effects of drug use and the negative effects of compulsive drug use on reward function (Koob 2003).

In addition to these neural circuits, systems of neurotransmitters and neuromodulators^6^ such as GABA, opioid peptides, and endogenously formed cannabinoids (i.e., endocannabinoids [eCBs]) have been found to be involved in the acute reinforcing effects of drug use. Researchers recently have identified interactions between alcohol, eCBs, and opioids. Specifically, these interactions affect transmissions of GABA that depress the activity of the cell on which they act (i.e., inhibitory synaptic transmission).^7^ Numerous sensory neurons, and most of the neurons that connect sensory neurons to each other (i.e., interneurons), contain GABA and control local synaptic networks, suggesting that GABA is a critical regulator of the transmission of nerve signals. Because opioids, cannabinoids, and alcohol act on the same transmitter (GABA) in the same brain regions (the CeA), dissecting these drug interactions on a common cellular target could uncover a key neuroadaptative site and cellular mechanisms triggered by abused substances. This article reviews the actions and interactions of alcohol and opioid and cannabinoid substances on the amygdalar GABAergic system.

Fine-tuning the GABAergic inhibitory system in the CeA is a prerequisite for controlling the neurons that transmit to the nuclei downstream of the CeA. Most of the CeA neurons are GABAergic (Cassell et al. 1999; Sun and Cassell 1993) and are either inhibitory neurons with recurrent or feedforward connections^8^ or inhibitory neurons that project to the brainstem or BNST targets. The CeA can be thought of as a “gate” that regulates the flow of information through the intra-amygdaloidal circuits, leading to changes in the inhibition of downstream regions. The following section will discuss how alcohol affects the GABAergic system and how these alterations may functionally influence the neuronal responsivity of the inhibitory CeA gating. In addition to the modulation of GABAergic transmission by alcohol, opioids, and cannabinoids, this article also will describe direct interactions that take place among these drugs. Research ultimately may be able to more directly address the cellular and synaptic neuroadaptations associated with the development of drug addiction. Understanding the cross-talk (i.e., nerve cell communication) between these systems may be critical for the development of new treatments for drug addiction and abuse and also may be helpful for combination therapy (Cichewicz 2004*a*).

## Alcohol’s Actions on GABA Transmission

The most consistent effect of alcohol on the brain is to reduce neuronal activity by a combination of effects that increases the inhibitory action of GABA and decreases the excitatory action of the neurotransmitter glutamate. GABAergic transmission is sensitive to alcohol in several brain regions and is involved in the acute actions of alcohol as well as long-term effects, such as the development of tolerance and dependence (Criswell and Breese 2005; Siggins et al. 2005; Weiner and Valenzuela 2006). To better define the potential interactions taking place on the amygdalar GABAergic system, it is important to know how alcohol alters GABA transmission and, in turn, how GABAergic activity modulates AOD-seeking behaviors. Behavioral studies have implicated GABAergic transmission in regulating alcohol intake. The reinforcing effect of alcohol is prevented by administering agents into the CeA that block receptors (i.e., receptor antagonists) for GABA_A_ (a subtype of GABA receptor) and opioids (Heyser et al. 1999; Hyytia and Koob 1995). Other studies (Merlo Pich et al. 1995; Roberts et al. 1996) found that alcohol-dependent rats appeared to have enhanced sensitivity to agents that mimic GABA_A_ receptors (i.e., receptor agonists). Furthermore, activation of GABA_A_ receptors in the CeA decreases alcohol self-administration only in alcohol-dependent rats (Roberts et al. 1996), indicating increased GABAergic transmission in alcohol reinforcement and dependence.

**Figure 1:**
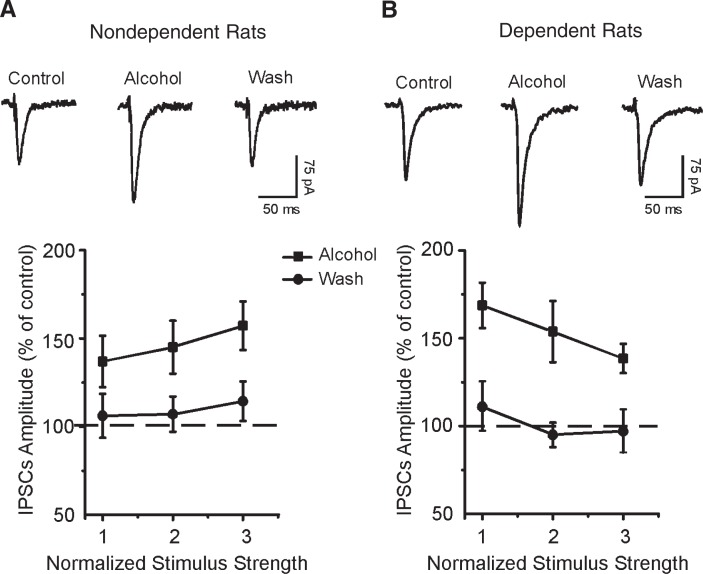
Alcohol enhances transmission of the neurotransmitter γ-aminobutyric acid (GABA) in the central nucleus of the amygdala (CeA). **A)** Top: Representative recordings of GABA-mediated inhibitory postsynaptic currents (IPSCs) in a CeA slice from a non–alcohol-dependent rat recorded before, during, and after the slice was exposed to alcohol (i.e., superfused*). Bottom: Pooled data showing that superfusion of alcohol (44 mM) significantly increased the average IPSC amplitudes in CeA neurons from non–alcohol-dependent rats. **B)** Top: IPSC recordings in a CeA slice from an alcohol-dependent rat. Bottom: Alcohol significantly increased the mean IPSC amplitudes in alcohol-dependent rats. NOTES: The CeA is one of a group of brain structures that plays an important role in both the acute reinforcing effects of drug use and the negative effects of compulsive drug administration on the reward function part of the drug reward system. IPSCs reflect the transmission of chemical signals between neurons across microscopic gaps called synaptic clefts. A neuron sending a signal (i.e., a presynaptic neuron) releases a neurotransmitter, which binds to a receptor on the surface of the receiving (i.e., post-synaptic) neuron. Inhibitory neurotransmitters, such as GABA, depress the activity of the postsynaptic cell. Error bars (on A and B, bottom) represent standard error. *Superfusion is to flush a fluid over the surface of a tissue. SOURCE: Roberto et al. 2003, 2004.

Research at the cellular level implicates GABA in the intoxicating effects of alcohol (Martz et al. 1983). In studies of CeA samples from rats, Roberto and colleagues (2003) showed that the increase in GABAergic transmission is related to the dose, or amount, of alcohol supplied (see figure 1), which acts principally at a presynaptic site to augment GABA release. Furthermore, Roberto and colleagues (2004) confirmed the presynaptic effect of alcohol on GABA release in an in vivo microdialysis study that showed increased dialysate^9^ GABA levels in the CeA when alcohol was infused (Roberto et al. 2004). The investigators also assessed whether GABAergic synaptic changes occur with alcohol dependence in rat CeA. In CeA neurons from dependent rats, basal GABAergic transmission (via increased tonic GABA release^10^) was significantly higher than in non–alcohol-dependent rats. Moreover, acute alcohol still increased GABAergic transmission (see figure 1), suggesting a lack of tolerance to the acute effects of alcohol (Roberto et al. 2004). In addition, the in vivo data showed a four-fold increase of baseline dialysate GABA concentrations in the CeA of alcohol-dependent rats compared with non-dependent rats and confirmed the lack of tolerance to the acute local administration of alcohol by increased dialysate GABA levels in alcohol-dependent rats.

**Figure 2:**
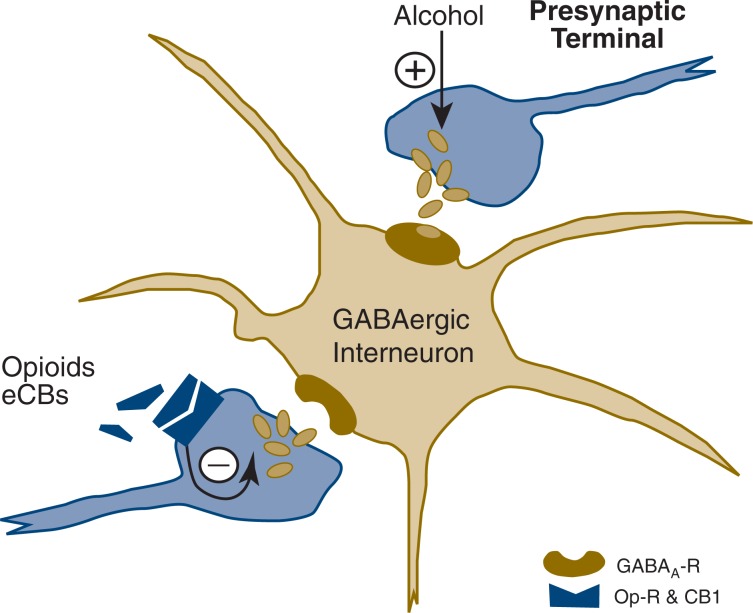
Hypothetical action of alcohol on γ-aminobutyric acid (GABA)-releasing synapses in the central nucleus of the amygdala. Upper synapse: Alcohol enhances the release of GABA from the presynaptic terminals of the same or another GABAergic interneuron. Lower synapse: Activation of presynaptic opioid or cannabinoid receptors (e.g., CB1) may reduce GABA release onto this interneuron, leading to increased excitability. NOTES: CB1, cannabinoid receptor; eCBs, endocannabinoids; GABA_A_-R, GABA_A_ receptor; Op-R, opioid receptor.

In summary, the findings of Roberto and colleagues (2004) point to a significant effect of alcohol on GABAergic transmission in the CeA that markedly adapts during the development of alcohol dependence. The mechanism of alcohol’s effect remains to be fully elucidated but primarily is associated with an action upon GABA release. Most of the CeA neurons used in this research are GABAergic inhibitory interneurons with inhibitory recurrent or feedforward connections as well as projections to downstream nuclei. Functionally, alcohol itself may influence the neuronal responsivity of the inhibitory CeA gating that regulates information flow through the intra-amygdaloidal circuits (e.g., by disinhibition of CeA), altering the inhibition of the downstream regions (e.g., BNST) by altering GABA release there (see figure 2).

## The Opioid System

Opioids decrease both excitatory (glutamatergic) and inhibitory (GABAergic) synaptic transmission (Siggins et al. 2003; Williams et al. 2001). The opioid-induced decrease of GABAergic transmission can occur via inhibition of GABAergic interneurons and GABA release, resulting in the disinhibition of descending neurons^11^ in a circuit. Via this disinhibition, opioids have excitatory actions in multiple regions of the nervous system in vivo, despite their inhibitory effects at the cellular level (Charles and Hales 2004). This mechanism mediates the pain-relieving effects of opioids (Christie et al. 2000) as well as the effects involved in opioid dependence (Williams et al. 2001; Xi and Stein 2002). In the brain region known as the ventral tegmental area (VTA), opioids bind to the receptor subtypes μ- and perhaps δ-receptors found on GABA interneurons or GABA projection neurons that interact with dopaminergic neurons, causing disinhibition of dopaminergic cell firing (Wise 1998). Although all the major types of opioid receptors are present in the NAcc, opioid actions have not been well characterized and may even be independent of the dopamine system in this brain region. In the NAcc, opioids inhibit the release of GABA at synapses between medium spiny neurons^12^ and interneurons, causing decreased inhibitory responses (Chieng and Williams 1998).

The CeA has one of the highest immunoreactive densities of enkephalins^13^ in the brain. Enkephalin is localized in the cell bodies of GABAergic neurons, the most abundant type of neuron in the CeA (Veinante et al. 1997). Moderate levels of μ-receptors (i.e., a subtype of opioid receptors) are found in the CeA (Chieng et al. 2006), where their activation decreases excitatory and inhibitory transmission via a presynaptic action (Finnegan et al. 2005).

In addition, activation of opioid receptors in the CeA reportedly opens ion channels (see sidebar) to inhibit CeA neurons (Chieng et al. 2006). Zhu and Lovinger (2005) suggested that the μ-receptors are the only functional opioid receptors localized on the glutamatergic projections to the CeA, and their activation leads to an inhibition of glutamatergic transmission. Another study (Finnegan et al. 2005) found that presynaptic μ-receptors localized on the neurons carrying impulses to the CeA and projecting to a midbrain structure called the periaqueductal gray area decreased GABAergic but not glutamatergic transmission. Together, these findings indicate that μ-receptors can modulate release of both GABA and glutamate.

Preliminary data by the authors of this article indicate that μ-receptor– dependent modulation of neuronal properties, as well as GABA and glutamate transmission in the CeA, are complex and involve pre- and postsynaptic mechanisms. The mechanisms and the effects appear to be neuronal type- and circuitry-specific. Results also are now available from neurons in CeA slices taken from morphine-treated rats. These results show no significant changes in either the intrinsic neuronal properties or synaptic properties in morphine-treated animals compared with naïve animals. However, exposure to a μ-receptor antagonist via superfusion^14^ significantly increased the amplitude of GABAergic transmission in the CeAs of both chronic morphine-treated and naïve animals, suggesting tonic (i.e., constant) activation of μ-receptors in both conditions.

## The Endogenous Cannabinoid System

Cannabinoids act via a specific receptor (i.e., CB1) to alter central physiological processes such as cognition, locomotion, appetite, and pain (Iversen 2003). CB1s are among the most abundantly expressed neuronal receptors and are found throughout the brain, including the amygdala (Freund et al. 2003). The discovery of specific cannabinoid receptors led to the isolation of endogenously formed binding molecules (i.e., ligands), the eCBs. The principal eCBs, arachidonylethanolamide (anandamide) and 2-arachidonoylglycerol, are derived from fat molecules (i.e., lipids). These compounds are synthesized and degraded by neurons (Piomelli 2003). CB1 agonists affect both inhibitory and excitatory synaptic transmission. The synthetic cannabinoid agonist WIN55212-2 (WIN) diminishes GABAergic and glutamatergic synaptic transmission in the NAcc and a region of the amygdala known as the basolateral amygdala (BLA) (Azad et al. 2003; Freund et al. 2003; Piomelli 2003). CB1 activation leads to presynaptic inhibition of GABAergic transmission in globus pallidus^15^ neurons and in the VTA. In the latter case, depression of the GABAergic inhibitory input of dopaminergic neurons likely increases their firing rate in vivo and consequently also increases dopamine release in the projection region of VTA neurons (e.g., NAcc).

**Figure 3:**
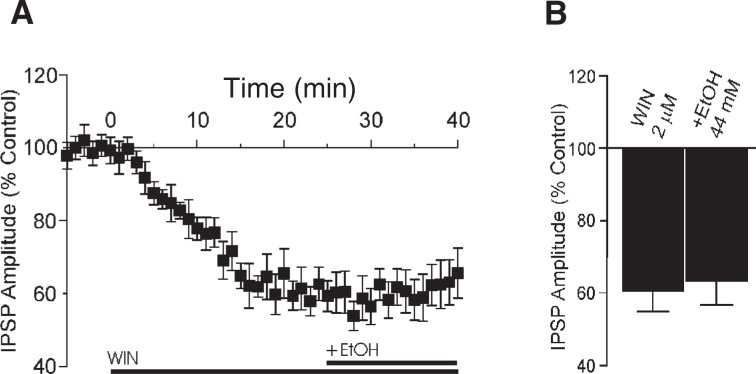
WIN, an agonist* for the cannabinoid receptor CB1, prevents alcohol from increasing transmission of the neurotransmitter γ-aminobutyric acid (GABA). Inhibitory synaptic responses were evoked in slices from the central nucleus of the amygdala (CeA) by locally stimulating the recorded neurons. **A)** Average of inhibitory postsynaptic currents (IPSC) amplitude over time. The application of 2 μM WIN (applied at *t* = 0) in the bathing media decreased IPSC amplitude. Further addition of 44 mM ethanol in the continued presence of WIN had no effect on CeA inhibitory transmission. **B)** On average (*n* = 7), 2 μM WIN decreased GABA transmission to 60 ± 5 percent of control (pre-WIN) values. The addition of 44 mM ethanol did not alter IPSC amplitude (63 ± 6% of control). *NOTE: For a definition of this and other technical terms, see the glossary pp. 177–179

In the BLA, eCB signaling has been implicated in the extinction of aversive memories (Marsicano et al. 2002). Zhu and Lovinger (2005) used freshly isolated BLA neurons to provide solid evidence of a retrograde eCB signaling^16^ in the BLA. Little information is available on the expression and function of CB1 in the CeA. Katona and colleagues (2001) reported that CB1 is expressed at high levels in certain amygdala nuclei, especially the lateral and basal nuclei, but are absent in the CeA. However, the authors recently conducted electrophysiological experiments in CeA slices to investigate the cellular interactions of the CB1 system and alcohol on GABAergic transmission (see below). These studies (Roberto et al., preliminary data) showed that superfusion of the CB1 agonist WIN onto CeA neurons markedly reduced GABA transmission (see figure 3).

Cannabinoids also affect neuronal excitability at the postsynaptic level. In the hippocampus, CB1 ligands close a family of potassium channels (i.e., M-channels) also affected by alcohol (Moore et al. 1990; Schweitzer 2000), and the concurrent modulation of M-channels by alcohol and cannabinoids could greatly affect neuronal excitability. Cannabinoids also open potassium channels of the G protein–regulated inwardly rectifying K^+^ channel (i.e., GIRK) type in transfected cells^17^ and have been linked to GIRK channels in the cerebellum. Interestingly, cannabinoid effects on synaptic transmission in the amygdala also appear to implicate GIRK channels (Azad et al. 2003), and GIRK conductances have been implicated in alcohol’s effects (Lewohl et al. 1999). Thus, M-channels and GIRK channels are potential sites of interaction between cannabinoids and alcohol at the postsynaptic level, and amygdala neurons display both M and GIRK currents (Meis and Pape 1998; Womble and Moises 1992).

Together, these results point to how the cannabinoid signaling system could be involved in some of the pharmacological effects of alcohol and a possible function for CB1 in alcohol tolerance and dependence, as discussed below. The eCB system may constitute part of a common brain pathway, mediating reinforcement of alcohol consumption and uncovering how the cannabinoid signaling system may provide a target for the treatment of alcoholism.

## Interaction of Alcohol and the Opioid System

Alcohol and opioid drugs have numerous behavioral effects in common, including sedation, motor depression, and rewarding experiences. Evidence exists to suggest that alcohol administration alters the release of endogenous opioid peptides (Gianoulakis 2004; Oswald and Wand 2004). Additional evidence indicates that an alcohol-induced increase of opioid peptide release affects alcohol consumption, and nonselective opioid antagonists that block all opioid receptors, such as naloxone and naltrexone, reliably decrease alcohol intake (Gianoulakis 2004; Oswald and Wand 2004).

### μ-Opioid Receptor

Pharmacological studies (Gianoulakis 2004; Oswald and Wand 2004) using subtype-specific antagonists show that the key element in opioid peptide systems involved in the positive reinforcing effects of alcohol is the μ-opioid receptor. Accordingly, μ-receptor antagonists dose-dependently reduce alcohol intake, and animals bred to have no μ-receptors have greatly diminished alcohol consumption and anxiety-like behaviors (Filliol et al. 2000; Roberts et al. 2000), suggesting an interaction between anxiety-like behavior and alcohol use.

### δ-Opioid Receptor

In contrast, mice lacking the δ-opioid receptor drink significantly more than controls (Roberts et al. 2001), possibly because of an increased level of anxiety in these animals. However, it also has been proposed that δ-receptors may be involved in the aversive properties of alcohol consumption, and thus it is possible that the increased alcohol intake in δ-receptor–deficient mice is produced by a diminution of the aversive effects of alcohol consumption. However, inconsistencies are found in the literature investigating the involvement of δ-receptors in alcohol preference and reward. For example, δ-receptor antagonists decrease alcohol consumption, and an inhibitor of the enzyme that breaks down enkephalins (i.e., enkephalinase) increases alcohol intake (Gianoulakis 2004; Oswald and Wand 2004), suggesting that δ-receptors exert a facilitatory influence on alcohol consumption.

### κ-Receptor

Considerably less information is available regarding the effect of κ-receptor ligands on alcohol self-administration. There is evidence that agonists for κ-receptors increase alcohol intake in Lewis rats (Lindholm et al. 2001), that alcohol-preferring rats have increased levels of the opioid prodynorphin in the thalamus and decreased κ-receptor densities in the hypothalamus compared with alcohol-avoiding rats (Marinelli et al. 1998), and that there are significantly higher levels of dynorphin peptides (κ-receptors agonists) in the NAcc of alcohol-avoiding animals compared with alcohol-preferring animals (Terenius 1994). Although inconsistencies exist in the literature, the cumulative evidence presently available suggests that alcohol consumption increases the release of endogenous opioid peptides and that μ- and δ-receptors facilitate the relationship between alcohol consumption and reward. Although relatively little information is available on the effect of κ-receptors in the regulation of alcohol consumption, it often is concluded that these receptors exert an inhibitory influence (for further review see Gianoulakis 2004; Herz 1997).

### Alcohol and Opioid Interactions in the CeA

In several brain regions, acute alcohol has been shown to release endogenous opioids, which, in turn, mediate alcohol effects, such as reinforcement and the reduction of anxiety (Oswald and Wand 2004). Consistently, acute alcohol administration increases expression of the gene *c-fos* (indicative of increased neuronal activity), specifically in the enkephalin-containing GABAergic neurons in the CeA (Morales et al. 1998). In addition, injection of opioid receptor antagonists into the CeA blocked the reinforcing effects of alcohol (Foster et al. 2004; Heyser et al. 1999).

Recent studies conducted in CeA slices showed that the alcohol-induced increase of GABAergic transmission was larger in mice without a functional δ-receptor gene (i.e., δ-receptor knockout mice). In addition, a δ-receptor inverse agonist, which binds to the same binding site as an agonist but exerts the opposite effect, augmented alcohol actions on GABAergic response in control mice to a level comparable with alcohol effects seen in δ-receptor knockout mice (Kang-Park et al. 2007). These findings suggest that endogenous opioids may reduce alcohol’s actions on GABA transmission in CeA neurons through selective δ-receptor–mediated inhibition of GABA release and that the increased alcohol effect on GABA responses in the CeA of δ-receptor knockout mice could, at least in part, be attributed to the absence of selective δ-receptor–mediated inhibition of GABA release. This result supports the hypothesis that endogenous opioid peptides modulate the alcohol-induced augmentation of GABA_A_ receptor–dependent circuitry in the CeA (Roberto et al. 2003). Ongoing studies suggest that baseline GABAergic transmission in the CeA is significantly greater in μ-receptor knockout mice compared with control mice. However, alcohol increased the GABAergic transmission to a similar extent in both μ-receptor knockout and control mice. It is possible that greater baseline activity of GABAergic transmission underlies the decreased anxiety-like behaviors and decreased voluntary alcohol consumption in μ-receptor knockout mice. This result is consistent with the suggestion that activation of GABA receptors in the CeA, possibly leading to stress reduction, is involved in mediating alcohol-seeking and drinking behavior.

## Interaction of Alcohol and the CB1 System

Chronic alcohol administration results in neurobiological alterations similar to those observed after chronic cannabinoid exposure (Mechoulam and Parker 2003). Recent behavioral and neurochemical evidence suggests that CeA eCBs are involved in alcohol dependence and the authors’ recent work points to interactions between alcohol and eCBs on synaptic transmission in the CeA. The neuroadaptation to chronic alcohol exposure has been shown to involve changes in the CB1 system, including alterations in the synthesis of eCBs and their precursors, as well as a decrease in the number of CB1 receptors (Basavarajappa and Hungund 2002; Gonzalez et al. 2004). It is important to explore the components of the eCB system in different brain areas to further understand how it may affect the development of alcohol dependence.

The eCBs acting at CB1 modulate alcohol consumption in rats, perhaps by affecting the activity of brain reward systems. The administration of a CB1 antagonist together with chronic alcohol treatment increases the preference for alcohol. In contrast, administration of a CB1 antagonist after chronic alcohol or at the time of withdrawal drastically diminishes alcohol preference and selectively reduces alcohol intake in alcohol-preferring rats (Serra et al. 2002). Similarly, acute administration of a CB1 antagonist suppresses only alcohol self-administration in alcohol-dependent animals, whereas operant responses^18^ for food are not affected. Further, chronic alcohol administration downregulates CB1 and increases the levels of the endogenous cannabinoid anandamide and the endogenous CB1 agonist 2-arachidonoylglycerol (Basavarajappa and Hungund 2002). These data suggest involvement of CB1 in alcohol reinforcement and a possible synergistic effect of eCBs in alcohol preference.

Cannabinoid ligands decrease GABAergic transmission, and research by the authors has shown that alcohol augments GABAergic transmission in the CeA, thus acting in a direction opposite of cannabinoids. A reciprocal influence to modulate synaptic transmission may therefore take place at inhibitory synapses. These results suggest that CB1 helps to regulate alcohol’s effects in CeA neurons. Superfusion of the CB1 agonist WIN inhibited evoked GABA transmission (i.e., GABA transmission that was evoked by stimulation of neurons) and completely blocked alcohol-induced effects (see figure 3), and the WIN effect was prevented by a selective CB1 antagonist (not shown). Thus, the selective activation of CB1 interferes with alcohol’s effects. Cannabinoids, by activating CB1 to decrease GABA transmission in CeA neurons, will diminish alcohol’s effects on the GABA system.

In summary, several lines of evidence designate the eCB system as a key player in the central effects of alcohol. (1) Blockade of CB1 drastically diminishes alcohol preference, suggesting that endogenous CB1 ligands reinforce the preference for alcohol; (2) chronic alcohol exposure increases the levels of eCBs; (3) CB1 may play a critical role in stress-stimulated alcohol use; (4) CB1 antagonists suppress alcohol self-administration but only in alcohol-dependent animals; and (5) chronic alcohol downregulates CB1 and increases eCB levels.

## Interaction of the Opioid and Cannabinoid Systems

Interactions between the opioid and cannabinoid systems influence specific and, very often, reciprocal cross-talks regulating many aspects of drug addiction (e.g. reward, dependence, tolerance, sensitization, and relapse) (Fattore et al. 2005, 2007). Behavioral evidence supporting interactions of opioid and cannabinoid systems in drug addiction includes (1) the blockade of some effects of the psychoactive compound Δ-9-tetrahydrocannabinol (THC) by opioid antagonists, (2) suppression of opioid withdrawal signs by cannabinoids and induction of withdrawal symptoms in morphine-dependent rats by CB1 antagonists, (3) precipitation of abstinence symptoms in THC-tolerant animals by naloxone, and (4) involvement of the cannabinoid and opioid systems in mechanisms underlying relapse (Cichewicz 2004*b*; Fattore et al. 2004, 2005, 2007). At the molecular level, studies (Caille et al. 2007; Vigano et al. 2005) have shown (1) co-localization of CB1 and opioid receptors in various brain regions, (2) alterations of the endogenous opioid system by exposure to cannabinoids and reciprocal alteration of the endogenous cannabinoid system by exposure to opioids, and (3) inhibition of cannabinoid stimulation of dopamine release in the NAcc by opioid antagonists. The mechanisms implicated in the interactions between opioids and cannabinoids involve several components of these systems, including alteration of the levels of endogenous ligands as well as their respective receptors. In addition, interactions between cannabinoids and opioids can trigger the modulation of other transmitter systems, such as GABA, glutamate, dopamine, and corticotropin-releasing factor (CRF).

The reciprocal alteration of endogenous levels of cannabinoids and opioids occurs in both acute and chronic states. Acute cannabinoid administration increases extracellular levels of endogenous dynorphin in the spinal cord (Houser et al. 2000; Mason et al. 1999; Pugh et al. 1997; Welch and Eads 1999) and enkephalin in the NAcc (Valverde et al. 2001). Similarly, chronic cannabinoid treatment increases gene expression of prodynorphin (which is a precursor to dynorphin A and dynorphin B, both additional opioids) and the opioid proenkephalin in brain regions mediating pain relief and drug dependence (Corchero et al. 1997*a*, *b*; Manzanares et al. 1998). Reciprocally, heroin self-administration into the NAcc significantly increases anandamide and decreases levels of the eCB 2-arachi-donylglycerol in this brain region (Caille et al. 2007). In addition, acute morphine increases anandamide levels in the hippocampus, NAcc, and caudate putamen and decreases 2-arachidonylglycerol levels in the NAcc and hippocampus (Vigano et al. 2004), whereas chronic morphine markedly lowers 2-arachidonylglycerol content without changing anandamide levels in these brain regions (Vigano et al. 2003, 2004).

**Nerve Cell Communication**

Nerve cells, or neurons, allow different parts of the body to communicate with each other by receiving, conducting, and transmitting electrical charges (i.e., impulses) throughout the nervous system. Chemical messengers called neurotransmitters carry information across small gaps or synapses that exist between neurons. A neuron sending a signal (i.e., a presynaptic neuron) releases a neurotransmitter, which binds to a receptor on the surface of the receiving (i.e., postsynaptic) neuron. Receptors are proteins with unique shapes that fit a specific neurotransmitter, much like a lock and key. For example, serotonin will only bind to serotonin receptors, not dopamine receptors.

The mechanism underlying neuronal signal transmission is based on voltage differences (i.e., potentials) that exist between the inside and the outside of the cell. This membrane potential is created by the uneven distribution of electrically charged particles, or ions, such as sodium (Na^+^) and potassium (K^+^). Ions enter and exit the cell through specific channels in the cell’s membrane (i.e., ion channels). The channels “open” or “close” in response to neurotransmitters or to changes in the cell’s membrane potential. Ligand-gated ion channels regulate ion passage through interactions with neurotransmitters. Voltage-gated ion channels open and close in response to changes in membrane potential and are named for the ion (e.g., potassium) that passes through its pore. The resulting redistribution of electric charge may alter the voltage difference across the membrane. A decrease in the voltage difference is called depolarization. If depolarization exceeds a certain threshold, an impulse (i.e., action potential) will travel along the neuron. The generation of an action potential is sometimes referred to as “firing.”

Depolarization of a presynaptic neuron results in the release of neurotransmitters into the synapse. Glutamate is the predominant excitatory neurotransmitter that promotes depolarization of a postsynaptic neuron, propagating an electrical signal. γ-Aminobutyric acid (GABA) is the major inhibitory neurotransmitter that blocks depolarization of a postsynaptic neuron. A neuron that makes a particular neurotransmitter, and therefore has the potential to release it, is designated by the neurotransmitter name with the suffix –ergic (e.g., GABAergic).

**Figure:**
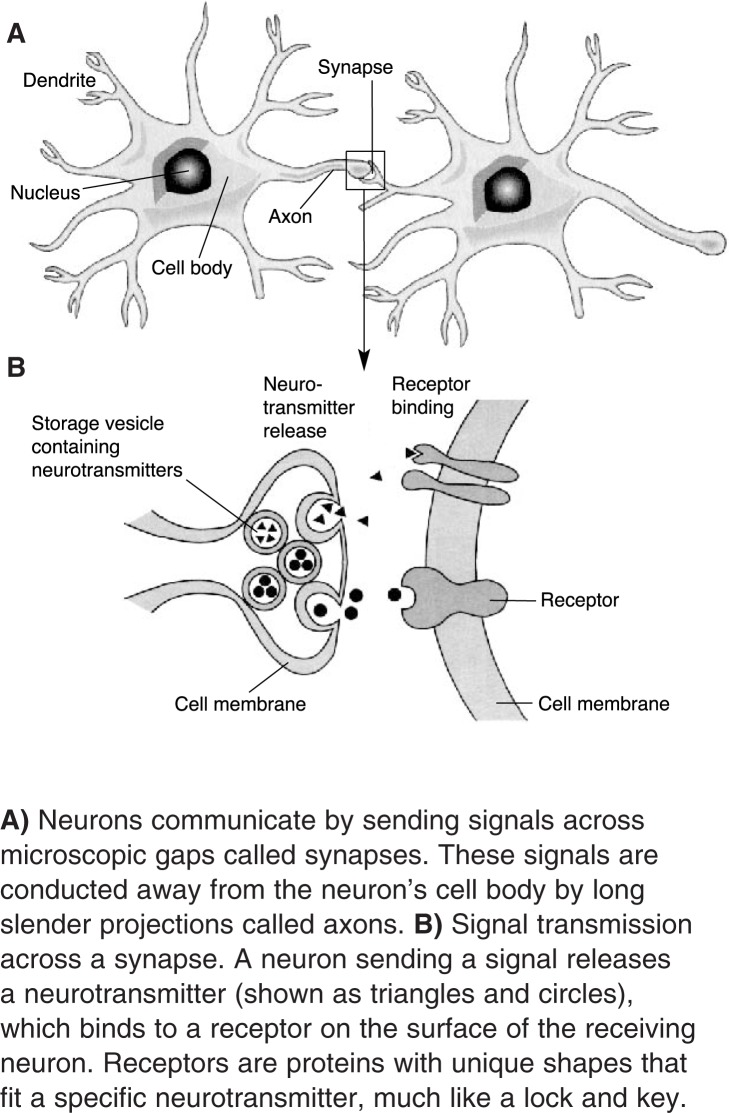
**A)** Neurons communicate by sending signals across microscopic gaps called synapses. These signals are conducted away from the neuron’s cell body by long slender projections called axons. **B)** Signal transmission across a synapse. A neuron sending a signal releases a neurotransmitter (shown as triangles and circles), which binds to a receptor on the surface of the receiving neuron. Receptors are proteins with unique shapes that fit a specific neurotransmitter, much like a lock and key.

Interactions of the opioid and cannabinoid systems at the receptor level have been observed mainly after chronic morphine treatment. In various studies, chronic morphine produced divergent effects as well as brain region–dependent effects on CB1 binding and expression levels. These studies have shown no change (Romero et al. 1998; Thorat and Bhargava 1994), decreased CB1 binding and expression in the cerebellum and hippocampus (Vigano et al. 2003), and increased CB1 binding in the caudate-putamen and limbic structures (Gonzalez et al. 2002). Gonzalez and colleagues (2003) reported decreased CB1 binding in the midbrain and cerebral cortex of morphine-dependent rats. In cannabinoid-tolerant animals, studies show increased μ-opioid receptor density in several brain regions (Corchero et al. 2004). In the core of the NAcc, researchers have suggested the allosteric (at a site other than the active site) binding of μ-opioid receptor and CB1 (Schoffelmeer et al. 2006). This study also showed that activation of these receptors resulted either in an excitatory (i.e., glutamate release) or inhibitory (i.e., GABA release) effect.

In summary, acute administration of cannabinoids and opioids elicits cellular interactions, principally altering the levels of the respective endogenous ligands and subsequent activation or inhibition of the relevant receptors. However, prolonged exposure to opioids and cannabinoids can trigger sustained interactions between these systems that may lead to neuroadaptations taking place at all levels of these endogenous transmitter systems, including ligand concentration and receptor density. How neuronal activity actually is affected by these interactions remains to be determined.

## Conclusion

The combined evidence now available clearly establishes the vast progress recently made in our understanding of the brain synaptic circuitry implicated in the transition to drug dependence. In an attempt to uncover a common site of cellular action for abused substances, the authors focused on the GABAergic system, a critical modulator of local synaptic activity in the CeA, a brain region considered to be a key element of the brain reward system. This review focuses on alcohol, opioids, and cannabinoids because these drugs only recently have been shown to interact at GABAergic synapses in the CeA. In addition, the authors are familiar with these drugs and were able to report on data generated in their laboratories. Because there are no receptors or well-defined transduction mechanisms for alcohol, the authors emphasize the cellular mechanisms affected by alcohol. Other drugs of abuse such as psychostimulants (i.e., amphetamine, cocaine) and nicotine are not discussed here but are described in other recent reviews (DiFranza and Wellman 2007; Kalivas 2007; Rebec 2006).

Because the CeA is involved in alcohol reinforcement and dependence, the authors envision that the influence of opioids and eCBs to block the facilitatory effects of alcohol on GABAergic transmission in the CeA is altered in dependent rats. Our hypothesis is that alcohol consumption and dependence stimulates the release of endogenous cannabinoids and/or opioids, or stimulates these endogenous systems that function as “brakes” and limit the enhancement of GABAergic transmission by alcohol in the neural circuitry involved in reinforcement. Future studies assessing the cellular actions of opioid and cannabinoid transmitters in the CeA and their modulation of alcohol’s effects will likely lead to a better understanding of the cellular mechanisms of drug addiction. The endogenous opioid and cannabinoid transmitter systems are emerging as promising targets for future medications to treat drug dependence.

## Footnotes

1 Cannabinoids are a group of substances that are structurally related to tetrahydrocannabinol (THC, the active substance in marijuana) or that bind to cannabinoid receptors.

2 A chemical substance that transmits impulses between nerve cells (i.e., neurons).

3 The amygdalae (singular amygdala) are almond-shaped groups of neurons located deep within the medial temporal lobes of the brain. They encompass several nuclei, or structures in the central nervous system, including the central, lateral, and basal nuclei.

4 The nucleus accumbens is a group of neurons located in the forebrain that is thought to play a role in reward, pleasure, addiction, and fear.

5 The bed nucleus of stria terminalis is an area of the hypothalamus.

6 Neuromodulators are similar to neurotransmitters and change the way neurons react to those neurotransmitters.

7 Communication among neurons typically occurs across microscopic gaps called synaptic clefts. A neuron sending a signal (i.e., a presynaptic neuron) releases a neurotransmitter, which binds to a receptor on the surface of the receiving (i.e., postsynaptic) neuron. Excitatory neurotransmitters activate the postsynaptic cell and inhibitory neurotransmitters depress the activity of the postsynaptic cell.

8 Feedforward connections maintain a condition in its current state rather than allowing a change in the system.

9 Microdialysis is a technique used to analyze the chemical components of tissues. A solution (i.e., the dialysate) is pumped through a semipermeable membrane inserted into the tissue. Molecules in the tissue diffuse into the dialysate as it is pumped through the membrane and the dialysate is then collected and analyzed to determine the identities and concentrations of molecules that were in the tissue.

10 Tonic release means that GABA constantly is being released from the terminals, even without electrical stimulation. In dependent animals, tonic release is “increased,” meaning that the amount of GABA released is greater than in nondependent animals.

11 Descending neurons are neurons that carry information from the brain to the chest or abdominal region.

12 Medium spiny neurons are inhibitory and play a key role in initiating controlling movements of the body, limbs, and eyes.

13 Enkaphalins are endogenous compounds that bind to opioid receptors and are involved in regulating pain.

14 Superfusion is to flush a fluid over the surface of a tissue.

15 The globus pallidus is a structure in the brain involved in the regulation of voluntary movements at a subconscious level

16 Retrograde signaling is a phenomenon in which a signal travels from a postsynaptic neuron to a presynaptic one.

17 Transfected cells are cells into which foreign DNA has been inserted. In this case, the cells were manipulated to express GIRK channels.

18 A operant response is a behavior that is modifiable by its consequences.
